# Human vaccine candidates for infections caused by *Klebsiella pneumoniae*: A systematic review

**DOI:** 10.1002/hsr2.70061

**Published:** 2024-09-10

**Authors:** Hajar Motamed, Farideh Yari, Etrat Javadirad, Sima Golmohammadi, Saeed Alimoradi, Ronak Naleini, Roya Chegene Lorestani, Fatemeh Nemati Zargaran, Mosayeb Rostamian

**Affiliations:** ^1^ Clinical Research Development Center, Imam Reza Hospital Kermanshah University of Medical Sciences Kermanshah Iran; ^2^ Clinical Research Development Center, Imam Khomeini and Mohammad Kermanshahi and Farabi and Imam Reza Hospitals Kermanshah University of Medical Sciences Kermanshah Iran; ^3^ Department of Internal Medicine, School of Medicine Kermanshah University of Medical Sciences Kermanshah Iran; ^4^ Clinical Research Development Center, Taleghani and Imam Ali Hospital Kermanshah University of Medical Sciences Kermanshah Iran; ^5^ Hematopoietic Stem Cell Research Center Shahid Beheshti University of Medical Sciences Tehran Iran; ^6^ Infectious Diseases Research Center, Health Institute, Imam Reza Hospital Kermanshah University of Medical Sciences Kermanshah Iran; ^7^ Student Research Committee Kermanshah University of Medical Sciences Kermanshah Iran

**Keywords:** clinical trials, human vaccine, immune response, infection, *Klebsiella pneumoniae*, systematic review

## Abstract

**Background and Aims:**

There are many difficulties in treating *Klebsiella pneumoniae*, necessitating the creation of more preventative/therapeutic measures like vaccinations. However, after numerous attempts, there is still no authorized and widely accessible vaccine. The present study aimed to systematically review published studies on *K. pneumoniae* vaccines in human subjects/samples.

**Methods:**

To find published studies, several electronic databases, including Scopus, PubMed, Web of Science, ClinicalKey, ClinicalTrials.gov, and Cochrane Library were searched without time limitation using the appropriate keywords. Studies were scrutinized, and the information from those that met our inclusion criteria was gathered and analyzed.

**Results:**

In total, 691 studies were found, of which 14 articles were included for systematic review. Bacterial lysate containing *K. pneumoniae* was the most studied vaccine candidate. As the main indicator of human immune responses to *K. pneumoniae*, antibody responses were determined by most studies. The antigen amount, the route of immunization, and the immunization schedule were varying in the studies and were chosen based on several factors such as the disease model, the vaccine type, the vaccination setting (prophylactic or therapeutic), and so on.

**Conclusion:**

The majority of studies asserted that their vaccination was efficient and safe, which was demonstrated by a decrease in the rate of infections and the induction of protective antibody, cell‐dependent, and/or cytokine responses. Altogether, the information provided here will help researchers examine the *K. pneumoniae* vaccine candidates more closely and take future actions that will be more consistently successful.

## 1 INTRODUCTION

*Klebsiella pneumoniae* is a significant opportunistic pathogen causing infections in the respiratory tract, urinary tract, circulatory system, and wounds, particularly in individuals with underlying medical conditions.^1^, ^2^, ^3^, ^4^ Concerning the high mortality rate, a variety of health care is vital for sufferers infected with *K. pneumoniae*.^5^

The beta‐lactam resistance of *K. pneumoniae* has been expanded, basically due to the generation of the beta‐lactamase.^6^ Antimicrobials such as fluoroquinolones and carbapenems are endorsed to treat infections caused by beta‐lactam‐resistant *K. pneumoniae*, but there are expanding reports on resistance to these antibiotics as well.^7^ In addition to the restrained effect of antibiotics, there are some more challenges to treating *K. pneumoniae* infections including the phagocytosis prevention via the encapsulation of microorganisms, and the production of toxic shock‐causing endotoxins.^5^ These issues need creating suitable prevention programs such as vaccination, at least for people that are at risk.^8^

To now, a variety of vaccines, including killed or whole‐cell mutant, conjugate/polysaccharide, outer membrane proteins, lipopolysaccharide (LPS), fimbriae, and so on, have been studied for *K. pneumoniae* prevention in animals and/or humans.^8^ Also, there are some clinical trials aiming to prevent infections caused by *K. pneumoniae* alone or by several bacteria including *K. pneumoniae*.^8^ Despite these efforts, there is still no completely effective, authorized vaccine for *K. pneumoniae* that is also readily accessible.

We have recently conducted a systematic review on animal studies of *K. pneumoniae* vaccine candidates.^9^ Also, there are a few relevant reviews on *K. pneumoniae* vaccination in general.^10^, ^11^ However, there is no systematic review of the literature on *K. pneumoniae* vaccine candidates tested in humans. Therefore, the objective of this study was to thoroughly analyze all available information on *K. pneumoniae* vaccine candidates that have been tested on humans.

## 2 METHODS

### 2.1 Searching approach

Without time limitation (until July 7, 2022), the electronic databases of Scopus, PubMed, Web of Science, ClinicalKey, ClinicalTrials.gov, and Cochrane Library were searched to find published research on human vaccinations against *K. pneumoniae*. The following keywords were used: “*Klebsiella pneumonia*e,” “*K. pneumoniae*,” “*Klebsiella* spp.,” “pneumoniae,” “sepsis,” “respiratory tract infection,” “urinary tract infection,” “vaccination,” “vaccine,” “vaccine candidate,” “immunization,” “human,” “clinical trial,” “protection,” “immunogenicity,” “immunity,” “antigen,” “antibody,” and “cytokine” alone or combined using appropriate operators such as “AND” and/or “OR.” The investigation was carried out in accordance with the PRISMA (preferred reporting items for systematic reviews and meta‐analysis) criteria.^12^

### 2.2 Inclusion and exclusion criteria

The following were the inclusion criteria: (1) Research focused on developing vaccines (either therapeutic or prophylactic vaccines) to protect people from *K. pneumoniae* infections, (2) Clinical and preclinical trials, and experiments using human subjects or human samples, (3) Studies that used a *K. pneumoniae*‐derived vaccine, or a part of its vaccine, include *K. pneumoniae*, (4) Published studies in English.

The following were the exclusion criteria: (1) Animal studies and studies without human subjects or samples, (2) Narrative/systematic reviews, letters, books, conferences, and comments, (3) Non‐English, duplicate, and non‐full text available articles, (4) Research without experimental results (e.g., *in silico* studies), (5) Studies using auto‐vaccines that designed a vaccine based on the patients’ bacterial culture, hence their vaccine component varied accordingly.

### 2.3 Data collection

Several data were gathered from the selected studies as follows: authors, immunization setting, published year, country, infection's type, vaccine type, vaccine name, vaccine components, adjuvant, human population, sample type, sample size, control, gender, age, control, the amount of antigen used, route, schedule, immune response assay, safety, and the main outcomes. Two authors separately evaluated the articles’ quality, and MR then reviewed the results to resolve any inconsistencies.

## 3 RESULTS

**Figure 1:**
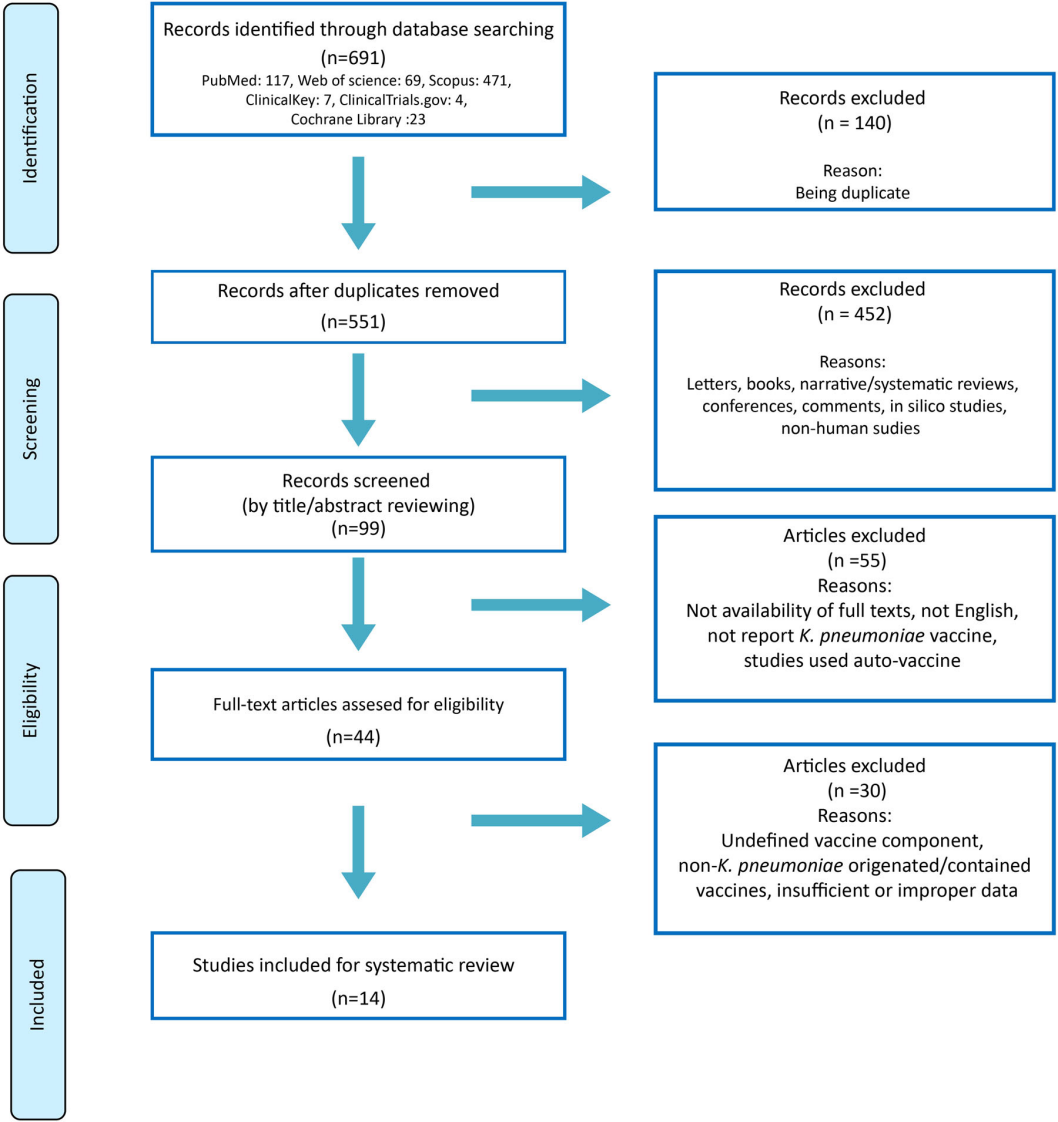
Flowchart of the study selection.

Following the search strategy, a total of 691 records was found, of which 140 were duplicates and hence were excluded. Of 551 remained studies, 452 records were excluded with reasons by title and abstract reading. Based on our exclusion criteria, 55 articles were excluded and 44 studies remained for eligibility assessment. After the eligibility step, 30 more studies were excluded and finally, 14 articles remained for systematic review (Figure 1).

### 3.1 General data

Of 14 included studies, seven were published before 2000, and seven were published after 2000. Four studies were performed in the USA, three studies were done in France and Spain, and one study was conducted in each Bulgaria, Portugal, Switzerland, and UK.

Individuals receive therapeutic vaccinations after they have already been exposed to the infection, whereas prophylactic vaccines are given to people as a preventative strategy to avoid the infection. In the present study, the setting was equal for prophylactic and therapeutic vaccination (seven studies for each).

The most infection/disease studied was recurrent urinary tract infection (UTI) (four studies), followed by respiratory tract infection (RTI) (two studies), acute trauma (one study), burn‐wound sepsis, periodontal disease (one study), and cancer (one study) (Table 1).

**Table 1:** The characteristics of human studies on *Klebsiella pneumoniae* vaccine candidates.

Setting	Authors	Published year	Country	Type of Infection/disease	Antigen type	Vaccine (antigen) name	Antigen components	Adjuvant	Population	Sample type	Control	Sample No.	Gender (No.)	Age (year)	Antigen amount	Route	Immunization schedule	Efficacy assessment	Is vaccine protective?	Is vaccine safe?	Main outcome	Reference
**Prophylactic vaccination**	Campbell et al.	1996	USA	Acute Trauma	Polysaccharide	Klebvax	24‐valent CPS	N/A	Victims of acute trauma	Sera	N/A	10	M (7), F (3)	18–44	1200 µg/patient	i.m	Day 0	Antibody response	Yes	N/A	Of 24 *Klebsiella* antigens, 9 patients responded to at least 18, and 7 patients responded to 22 antigens. One antigen, K35, was poorly immunogenic so that only 5 of 10 patients responded.	[13]
	Cryz et al.	1985	Switzerland	Burn‐Wound Sepsis	Polysaccharide	K1 CPS	Untreated or NaOH‐treated (to remove LPS) CPS	N/A	Healthy volunteers	N/A	N/A	N/A	Both genders	15–69	N/A	s.c	Day 0, 35	Antibody response	Partially	Yes	Immunization with either untreated or NaOH‐treated vaccines elicited a specific IgM and IgG response. However, by passive transfer of antibodies from vaccinated humans to mice no significant protection was observed	[14]
	Michel et al.	1987	France	N/A	Bacterial ribosomes	D53	Ribosomal fractions of *H. i*, *K.p*, *S.p*, and *S.py*	None	Healthy volunteers	Sera	Not immunized individuals	104 (51 case, 53 controls)	M	N/A	N/A	Aerosol	Series 1st: Week 0, 1, 2, 4, and 6 (3 times/week, 4 dose/each time). Series 2nd: Day 0 to 14, and month 6 (4 times/day, 2 dose/each time).	Antibody response	Yes	N/A	A significant difference was seen in the immunity of case and control groups. The specific antibodies increased with vaccination.	[15]
	Pedraza‐Sanchez et al.	2006	USA	N/A	Glycoprotein	RU 41740 (Biostim)	Capsular antigen and LPS	None	Healthy volunteers	PBMC	Cells with added PBS, LPS, or PHA	6	M (3), F (3)	20–46	0.05, 0.1, 1 or 5 µg/ml	Adding to cells	4 or 24 h after adding reagents	CD markers detection, Cytokine response	Yes	N/A	The vaccine induced CD80, CD69, CD 86, and IL‐12. The pro‐inflammatory cytokines (TNF‐a and IL‐6) were produced at 4 h and IFN‐g was induced at 5 h just in NK cells.	[16]
	Petrunov et al.	2006	Bulgaria	Periodontal disease	Bacterial lysate	Dentavax	A lysate of *S.py*, *K.p*, *S.a*, *C.a*, and *L.a*	None	Healthy volunteers	Sera, Saliva	N/A	12	M (3), F (9)	40–60	36 mg (each tablet)	Orally	Day 0 to 9 (6 tablet a day)	Antibody response, T cell response, Cytokine response	Yes	N/A	The early/late CD8 T cells ratio was increased. A significant effect of Dentavax was seen on both T and B cell activity. An increased level of specific antibodies against the components of Dentavax was found in both salivary and serum, with highest levels between days 7 and 21.	[17]
	Puigdollers et al.	1980	Spain	N/A	Bacterial lysate	Broncho‐Vaxom	A lysate of *D.p*, *H. i*, *K.p*, *K.oz*, *S.a*, *S.py, S.v*, and *N.c*	None	Healthy volunteers	Sera, Saliva	N/A	12	M (6), F (6)	15–44	7 mg (each capsule)	Orally	Day 0 to 9 (one capsule a day)	Antibody response	Yes	N/A	IgA in saliva was increased after 10 days. In sera, both IgM and IgG was increased (35 days and 5 months after immunization).	[18]
	Rytel et al.	1974	USA	RTI	Bacterial lysate	N/A	A heat‐killed lysate of *H. i*, *K.p*, *N.c*, *D.p*, Streptococci, and Staphylococci	None	Healthy volunteers	Sera, Throat sample	Placebo	95 (51 case, 44 control)	M	58.1 (mean)	N/A	s.c	Day 0, 7, 14, 21, Month 2, 3, 4, 5, 6, 7, 8, 9	Antibody response, Cytokine response, Clinical examination of individuals	No	N/A	No difference existed between the two groups in respiratory illness rates, hospital admissions, or clinic visits. No changes in normal throat flora were seen due to immunization. There was no interferon response after vaccination.	[19]
**Therapeutic vaccination**	Bene et al.	1993	France	N/A	Bacterial lysate	OM‐85 BV	A lysate of *D.p*, *H. i*, *K.p*, *K.oz*, *S.a*, *S.py, S.v*, and *N.c*	None	Children who required tonsillectomy	Tonsil sample	Placebo	28	N/A	3–16	3.5 mg	Orally	Day 0, 7	Antibody response	Partially	N/A	Slightly higher antibody response were seen after treatment with OM‐85 BV, but significant increases in antibody response were observed only for D53 group.	[20]
					Bacterial ribosomes	D53	Ribosomal fractions of *H. i*, *K.p*, *S.p*, and *S.py*	*K.p* proteoglycan				28			0.25 mg		Day 0, 7, 14		Yes			
	Latil et al.	1986	France	Cancer	Bacterial lysate	N/A	A lysate of *N. c*, *N.f*, *N. p*, *M. sp*, *M.p*, *K.p*, *H.i*, and *S.p* types I, II, III, V, VII, and XII	None	Patients who had undergone pneumectomy for cancer	Bronchi biopsy	Not immunized patients	22 (11 case, 11 control)	N/A	16–73	N/A	Aerosol	Day 0 to 9 (twice a day)	Antibody response	No	N/A	No *K. p* antibody‐bearing cells were found in either case or control group (in all epithelium, lamina propria, and submucous gland samples).	[21]
	Lorenzo‐Gomez et al.	2013	Spain	UTI	Bacterial lysate	Uromune	An inactivated bacterial cell suspension of *E.c*, *K.p*, *P.v*, and *E.f*.	None	Recurrent UTI patients	N/A	Patients treatd with SMX/TMP	319 (159 case, 160 controls)	F	16–87	2 puffs of 100 μl each (10^8^ bacteria/puff) daily	Sublingual	Day 1 to 30 (once a day)	Clinical examination of individuals	Yes	Yes	Compared to controls, patients in case group experienced a highly significant reduction in infection rate.	[22]
	Nickel et al.	2021	Spain	UTI	Bacterial lysate	Uromune	An inactivated bacterial cell suspension of *E.c*, *K.p*, *P.v*, and *E.f*.	None	Recurrent UTI patients	N/A	Women treated with antibiotic prophylaxis	1907 (1408 case, 499 control)	F	N/A	N/A	Sublingual	N/A	Assay T‐cell specific adaptive as well as innate immune response at the level of the genitourinary tract.	Yes	Yes	Women treated with Uromune had significantly lower UTI rates.	[23]
	Ruah et al.	2001	Portugal	RTI	Bacterial lysate	LW 50020	A lysate of *H. i*, *K.p*, *S.a*, *S.p*, *S.py*, *S.m*, and *B.c*	None	Recurrent RTI patients	N/A	Patients who received two active‐treatment cycles and two placebo cycles	154 (73 case, 81 control)	M (89), F (65)	4–12	N/A	Orally	Week 0‐3, 8‐11, 16‐19, 24‐27 (4 dose/week)	Clinical examination of individuals	Yes	Yes	In immunized indididuals the infection rate was reduced by 50% with the standard schedule (immunization cycle + one booster cycle) but could not be further reduced by two more boosters.	[24]
	Uehling et al.	2003	USA	UTI	Bacterial lysate	Urovac	A heat‐killed lysate of *E.f*, *K.p*, *P.mi*, *P.mo*, *E.c*	None	Recurrent UTI patients	Sera, urine, and cervical‐vaginal secretions	Placebo	54 (primary, booster, control group)	F	18–74	N/A	Vaginal	Week 0, 1, and 2 (primary group), Week 0, 1, 2, and month 2, 3, and 4 (booster group)	Antibody response, Clinical examination of individuals	Yes	Yes	Compared to women receiving placebo or primary immunizations, those receiving six doses of vaccine remained free of infections for a significantly longer period than. Of women receiving six doses 55% did not experience an infection, while 89% of placebo treated patients had UTIs.	[25]
	Yang et al.	2017	UK	UTI	Bacterial lysate	Uromune	An inactivated bacterial cell suspension of *E.c*, *K.p*, *P.v*, and *E.f*.	None	Recurrent UTI patients	N/A	None	75	F	18–87	N/A	Sublingual	Daily for 3 months	Clinical examination of individuals	Yes	N/A	Of the 75 patients who completed treatment, 78% had no subsequent UTIs. Among the patients with recurrent UTI, the urine culture of 12 were positive for *E. coli*. The remaining patients had mixed growth, Klebsiella, Pseudomonas, and *Serratia marcescens*.	[26]

Abbreviations: *B.c, Branhamella catarrhalis; C.a, Candida albicans*; CPS, capsule polysaccharide; *D. p, Diplococcus pneumoniae; E.c, Escherichia coli; E.f, Enterococcus faecalis*; F, female; *H.i, Haemophilus influenzae*; IL, interleukin; i.m, intramuscular; *K.p, Klebsiella pneumoniae; K.oz, Klebsiella ozaenae; L.a, Lactobacillus acidophilus*; LPS, lipopolysaccharide; M, male; *M.p, M. pyogenes; M.sp, Moraxella sp*; N/A, not available; *N.c, Neisseria catarrhalis; N.f, N. flava; N.p, N. perflava*; PBMC, periferal blood mononuclear cell; PBS, phosphate buffer saline; PHA, phytohematuglutinine; *P.mi, Proteus mirabilis; P. mo, Proteus morganii; P.v, Proteus vulgaris*; RTI, respiratory tract infections; *S.a, Staphylococcus aureus; s.c, subcutaneous; S.m, Streptococcus mitis; S.p, Streptococcus pneumoniae; S.py, Streptococcus pyogenes; S.v, Streptococcus viridans*; SMX/TMP, Sulfamethoxazole/Trimethoprim; UTI, urinary tract infections.

The most sample used was sera samples (six studies), followed by saliva samples (two studies), and one study of each of peripheral blood mononuclear cells (PBMCs), bronchus biopsy, cervical‐vaginal secretions, throat sample, tonsil sample, and urine.

### 3.2 Vaccines type/components

The most used vaccine type was bacterial lysate containing *K. pneumoniae* with 10 records. Other vaccine types include bacterial ribosomes (two records), polysaccharides (two records), and glycoprotein (one record). The bacterial lysate studies have used inactivated or killed lysate of several bacteria including *K. pneumoniae*. Accompanied with *K. pneumoniae*, these bacteria are all involved in the *K. pneumoniae*‐caused infections. Both records of bacterial ribosomes used ribosomal fractions of *K. pneumoniae*, *Haemophilus influenzae*, *Streptococcus pneumoniae*, and *Streptococcus pyogenes*. The study of glycoprotein and both studies of polysaccharides used capsular polysaccharide (CPS) as the main component of their vaccines. Adjuvant was used only in one study which applied *K. pneumoniae* proteoglycan as adjuvant beside bacterial ribosomes vaccine (Table 1).

### 3.3 Populations

The populations used differ based on the vaccination setting (prophylactic or therapeutic vaccination). In six out of seven prophylactic studies, healthy volunteers were vaccinated, while in one prophylactic study, victims of acute trauma were used. The mean sample size was 39.83, and mainly adults were used. In four prophylactic studies both genders were used, while in two studies only men were used.

In therapeutic studies, four studies were done on UTI patients, and one study was done for each of children who required tonsillectomy, patients who had undergone pneumectomy for cancer, and recurrent RTI patients. The mean sample size was 369.57 and the vast range of ages was used. The most therapeutic studies were done on women with recurrent UTI (four studies), while in one study both genders was used and the gender was unknown in two studies (Table 1).

### 3.4 Immunization

The amount of antigen for immunization was only reported in seven studies and was varying based on the vaccine type.

The used routes were varying based on the vaccine type and include orally (four studies), sublingual (three studies), aerosol (two studies), subcutaneous (s.c) (two studies), adding to cells (one study), intramuscular (i.m) (one study), and vaginal (one study) (Table 1).

Except one study, the booster dose/doses were used in the studies. The immunization schedule was varying based on the vaccination setting and the vaccines type (Table 1).

Antibody response had been assayed in the majority of studies (nine studies) as the main indicator of *K. pneumoniae*‐specific immune response. Other arms of immune response were also studied in some studies as follows: T cells activation (three records), cytokines (three records), and innate immune responses (one record) (Table 1).

### 3.5 Efficacy/effectiveness of used vaccines

The effects of *K. pneumoniae* vaccine candidates on human subjects/samples were determined by two factors, first, the immune response, and second the protection. The protection (i.e., the reduction in the infection rate and/or severity) was reported in seven studies in which five studies reported a relatively protective effect of their vaccine, while two studies did not observe protection in their vaccinated groups. This non‐protective effect was seen for K1 CPS (a polysaccharide vaccine) and a bacterial lysate of *K. pneumoniae*, *H. influenzae*, *Neisseria catarrhalis*, *Diplococcus pneumoniae*, Streptococci, and Staphylococci.

The immune response as another indicator of the vaccine efficacy was assayed in nine studies. The appropriate antibody response was observed in six studies, and the appropriate cell‐dependent/cytokine responses were reported in two studies. One study which used a bacterial lysate of *K. pneumoniae*, *H. influenza*, *N. catarrhalis*, *N. flava*, *N. perflava*, *Moraxella sp*., *M. pyogenes*, and *S. pneumoniae* did not observe specific antibody response. Also, a study that used bacterial lysate of *K. pneumoniae*, *H. influenzae*, *N. catarrhalis*, *D. pneumoniae*, Streptococci, and Staphylococci, did not observe appropriate cytokine (interferon) responses (Table 1).

### 3.6 Safety

The safety of used vaccines was only reported in five studies. No adverse effect was seen in any of these five studies, hence the vaccines were all safe (Table 1).

## 4 DISCUSSION

In the current study, we conducted a systematic review of all studies on human vaccines against *K. pneumoniae* infections. The number of studies that were included was 14 studies, which is a small quantity, particularly given the fact that there were no time limits. The relatively small number of studies may be attributed to the difficulty and expensive approaches to *K. pneumoniae* vaccination, the inadequate *K. pneumoniae* antigens as vaccine candidates, the safety issue, and several more challenges of *K. pneumoniae* vaccination. Two of several challenges in developing *K. pneumoniae* vaccines are the most significant ones; first, there should be no cross‐reactivity between the vaccine and the natural flora of the gut, where *K. pneumoniae* colonizes,^27^ Second, the majority of *K. pneumoniae*‐vulnerable individuals are older and have compromised immune systems as a result of various comorbidities, which has resulted in decreased or nonexistent vaccine protective responses.^28^

Previously, a few vaccines were developed and tested in humans.^8^ Therefore, it was expected that much more studies be found on humans, but surprisingly only 14 relevant studies were found in the databases. In addition to the limitations mentioned above about difficulties of *K. pneumoniae* vaccination, the low number of human studies may refer to the ineffectiveness of the previously‐introduced vaccines in afterward evaluations. It should be also noted that the sample size of many of these 14 human studies was low which undermine their obtained results. In addition, half of the human studies on *K. pneumoniae* vaccination were done before 2000, and only three studies were done in the last 10 years. This also shows the reluctance of researchers to conduct *K. pneumoniae* vaccination studies in humans. Considering the importance of *K. pneumoniae* vaccination (at least for people at risk), more favorable conditions should be provided to conduct much more studies in this field. It is noteworthy that some clinical trials are ongoing in this field that were not included in our study since their data is not fully available yet.

Out of 14 studies included in our systematic review, 10 studies used bacterial lysates, and eight of them showed degrees of protection against *K. pneumoniae*. Although whole cells vaccines are usually considered as main inducers of immune responses, their most significant challenge is the issue of safety, demanding new antigens and/or strategies to develop safer vaccines.^29^, ^30^ Other studies in our systematic review used ribosomes, polysaccharides, and glycoprotein.

The immune system's most powerful weapons against *K. pneumoniae* are antibodies, which may attach to its surface molecules, neutralize it, and facilitate phagocytosis.^31^ This mechanism is known as opsonization, by which phagocytes recognize the Fc region of the antibodies coating the pathogen.^32^ Because of this, most of the studies employed antibody responses as the primary measure of immunological responses. T cell‐dependent responses that are mediated by cytokines, in addition to antibody responses, are crucial for producing complete protection against *K. pneumoniae*, although their precise functions are still unclear.^10^

The antigen amount, the route of immunization, and the immunization schedule were varying in the studies. However, it was expected, because these characteristics depend on a number of variables, including the disease model, the vaccine type, the vaccination setting (prophylactic vs. therapeutic), and so on. As previously reported,^33^ infection dose is a determining factor that may alter the intensity or direction of immune responses, and as a result, alter the infection's final outcome. Therefore, if it is possible, it is recommended that a pilot study be performed before immunization to find the best dose and route.

The majority of our analyzed studies claimed a protective and safe *K. pneumoniae* vaccine candidate. However, since studies indicating inefficient, non‐protective, or hazardous vaccines tend to be more rejected by journals than studies with positive findings, publication bias may have occurred in this field, especially since most of the studies were published a long time ago, but no significant progress has been reported afterward, and none of them have been approved for use until now. The good news is that several of these studies present a potent and safe *K. pneumoniae* vaccine that is suggested to be further studied to potentially defeat this life‐threatening pathogen.

As a limitation of our study, it should be mentioned that we excluded books, letters, conferences, and comments. The most accurate *K. pneumoniae* vaccination studies were found with the use of this exclusion, but it may have left out some less well‐known but yet potent vaccine candidates.

## 5 CONCLUSION

Altogether, in the present study, we thoroughly examined all available information on vaccine candidates of *K. pneumoniae* tested in human. This information includes information on antigens, immunization setting and schedule, safety, and the results of earlier research. The gathered and well‐clustered data of the present systematic review would help researchers to evaluate the potential *K. pneumoniae* vaccines more thoroughly and to make a more fruitful future.

## AUTHOR CONTRIBUTIONS

**Hajar Motamed**: Methodology; Writing—original draft; Writing—review and editing; Investigation. **Farideh Yari**: Data curation. **Etrat Javadirad**: Writing—review and editing. **Sima Golmohammadi**: Writing—review and editing. **Saeed Alimoradi**: Writing—review and editing. **Ronak Naleini**: Writing—review and editing. **Roya Chegene Lorestani**: Data curation; Writing—review and editing. **Fatemeh Nemati Zargaran**: Data curation. **Mosayeb Rostamian**: Conceptualization; Data curation; Writing—review and editing.

## CONFLICT OF INTEREST STATEMENT

The authors declare no conflicts of interest.

## TRANSPARENCY STATEMENT

The lead author Mosayeb Rostamian affirms that this manuscript is an honest, accurate, and transparent account of the study being reported; that no important aspects of the study have been omitted; and that any discrepancies from the study as planned (and, if relevant, registered) have been explained.

## Data Availability

The data that support the findings of this study are available from the corresponding author upon reasonable request. The datasets used and/or analyzed during the current study are available from the corresponding author on reasonable request.
